# Multimorbidity of communicable and non-communicable diseases in low- and middle-income countries: A systematic review

**DOI:** 10.1177/26335565221112593

**Published:** 2022-09-01

**Authors:** Lucy Kaluvu, Ogechukwu Augustina Asogwa, Anna Marzà-Florensa, Catherine Kyobutungi, Naomi S Levitt, Daniel Boateng, Kerstin Klipstein-Grobusch

**Affiliations:** 1 Julius Global Health, Julius Center for Health Sciences and Primary Care, University Medical Center Utrecht, Utrecht University, Utrecht, Netherlands; 2 Faculty of Health, Social Care and Medicine, Edge Hill University, Ormskirk, UK; 3 Prinses Maxima Center for Pediatric Oncology, 8124 University Medical Center Utrecht, Utrecht, Netherlands; 4 107883 African Population and Health Research Center, Nairobi, Kenya; 5 Chronic Disease Initiative for Africa, Department of Medicine, Faculty of Health Sciences, 63726 University of Cape Town, Cape Town, South Africa; 6 Department of Epidemiology and Biostatistics, School of Public Health, Kwame Nkrumah University of Science and Technology, Kumasi, Ghana; 7 Divison of Epidemiology and Biostatistics, School of Public Health, Faculty of Health Sciences, University of the Witwatersrand, Johannesburg, South Africa

**Keywords:** multimorbidity, communicable diseases, non-communicable diseases, low- and middle-income countries, health systems

## Abstract

**Objective:**

The aim of this systematic review is to analyse existing evidence on prevalence, patterns, determinants, and healthcare challenges of communicable and non-communicable disease multimorbidity in low- and middle-income countries (LMICs).

**Methods:**

PubMed, Cochrane, and Embase databases were searched from 1^st^ January 2000 to 31^st^ July 2020. The National Institute of Health (NIH) quality assessment tool was used to critically appraise studies. Findings were summarized in a narrative synthesis. The review was registered with PROSPERO (CRD42019133453).

**Results:**

Of 3718 articles screened, 79 articles underwent a full text review of which 11 were included for narrative synthesis. Studies reported on 4 to 20 chronic communicable and non-communicable diseases; prevalence of multimorbidity ranged from 13% in a study conducted among 242,952 participants from 48 LMICS to 87% in a study conducted among 491 participants in South Africa. Multimorbidity was positively associated with older age, female sex, unemployment, and physical inactivity. Significantly higher odds of multimorbidity were noted among obese participants (OR 2.33; 95% CI: 2.19–2.48) and those who consumed alcohol (OR 1.44; 95% CI: 1.25–1.66). The most frequently occurring dyads and triads were HIV and hypertension (23.3%) and HIV, hypertension, and diabetes (63%), respectively. Women and participants from low wealth quintiles reported higher utilization of public healthcare facilities.

**Conclusion:**

The identification and prevention of risk factors and addressing evidence gaps in multimorbidity clustering is crucial to address the increasing communicable and non-communicable disease multimorbidity in LMICs. To identify communicable and non-communicable diseases trends over time and identify causal relationships, longitudinal studies are warranted.

## Introduction

Multimorbidity defined as the co-existence of two or more chronic conditions within an individual is a major public health challenge globally.^1^ In low- and middle-income countries (LMICs), communicable diseases such as human immunodeficiency virus (HIV) and tuberculosis (TB), as well as nutritional deficiencies and maternal and child complications have been the leading causes of morbidity and mortality.^1–3^ However, with ongoing demographic epidemiological transition facilitated by socioeconomic development, non-communicable diseases such as hypertension, type 2 diabetes, cardiovascular disease (CVD), and cancer have become more common among the old and young,^3,4^ contributing to reduced quality of life, increased functional limitations and disability, and early mortality.^1,5^ Consequently, the economies of LMICs have been affected owing to the reduced workforce.^6^

For HIV and TB-endemic countries such as South Africa, China, and India, a rise in the prevalence of non-communicable diseases such as obesity and CVD has been reported especially among females living with HIV in socioeconomically disadvantaged areas.^4^ With the establishment of anti-retroviral (ART) programs and an increased life expectancy and premature ageing, high prevalence of non-communicable diseases has contributed to a higher burden of communicable and non-communicable disease multimorbidity.^4,7^

In many LMICs, high mortality rates and an increased risk of developing multiple chronic conditions has resulted from exposure to behavioral factors such as unhealthy dietary habits, physical inactivity, and excessive alcohol and tobacco use.^8^ Studies conducted in high-income countries (HICs) have reported a higher prevalence of multimorbidity among the aging population.^1^ However, there is a dearth of evidence from LMICs on the prevalence of multimorbidity among the young age groups and those residing in socioeconomically disadvantaged areas.^1^

In many LMICs, care models are designed to address single morbidities as opposed to multimorbidity. Compared to those with single health conditions, individuals with multiple chronic conditions suffer more often higher rates of unplanned hospital admissions and readmissions, as well as an increased use of emergency services.^9,10^ Also, where out of pocket payment for healthcare is high, multimorbidities exert even more financial pressure on households and can be drivers of repeated catastrophic healthcare expenditure.

Many individuals with multimorbidity become economically and socially dependent on their support networks and communities.^9,11,12^ Owing to the complexity of multimorbidity care, patients are prescribed multiple medication which increases their risk of drug interactions. Subsequently, they also develop poor medication adherence.^12^ Most of these negative impacts of multimorbidity might even worsen in LMICs where majority of the healthcare systems are fragmented, and at the same time infrastructurally designed to address single chronic conditions.^1,13^ On the other hand, communicable disease management is central in most endemic LMICs; thus, the complexity of communicable diseases and non-communicable diseases management is evident.^4^ In addition, many healthcare systems in LMICs are not adequately funded and this has resulted in low health service coverage and limited access to health insurance particularly for multimorbidity patients and the elderly.^1,12^ Evidence points to the subsequent catastrophic expenditure where the financial implication of healthcare related out-of-pocket (OOP) payments has resulted in a continued cycle of poverty for many households and communities.^12^

To date, multimorbidity has been mainly researched in HICs mostly with a focus on multimorbidity of non-communicable diseases.^1,3,14^ A recent systematic review on multimorbidity conducted among LMICs focused on non-communicable diseases multimorbidity among adults in LMICs.^15^ Data on the burden, disease patterns, and determinants of communicable diseases and non-communicable disease multimorbidity and population groups most afflicted in LMIC is inadequate.^3,9^ This evidence, however, is critical for the integration of communicable diseases and non-communicable diseases disease management for policy and practice. Hence, the aim of this systematic review was to synthesize existing evidence on the prevalence, patterns, and determinants of multimorbidity of communicable diseases and non-communicable diseases in LMICs. In addition, management and healthcare challenges associated with communicable diseases and non-communicable diseases multimorbidity were assessed.

## Methods

We conducted a systematic review of multimorbidity of communicable diseases and non-communicable diseases in LMICs, with a focus on articles reporting on prevalence, patterns, determinants, and management of multimorbidity. The methodology and reporting of findings were guided by the 2009 PRISMA (Preferred Reporting Items for Systematic Reviews and Meta-Analyses) checklist^16^ (Supplementary file 1). The systematic review protocol was registered on PROSPERO under CRD42020162180. No deviations were made from our pre-registered protocol.

### Search strategy

MeSH terms used for the systematic review were: “comorbidity,” “multimorbidity,” “communicable diseases,” “non-communicable diseases,” and “low- and middle-income countries.” A comprehensive search was conducted in PubMed, Cochrane, and Embase, using a combination of MeSH and variation terms as title/abstract headings. Articles published in English from 1^st^ January 2000 to 31^st^ July 2020 were selected. We selected a cut-off year (the year 2000) and focused on the last 20 years of reported studies to collate the most recent evidence spanning two decades of communicable and non-communicable comorbidities and multimorbidity research. The full search strategy for PubMed and Embase is presented as Supplementary file 2.

### Multimorbidity definition

The term “multimorbidity” was defined as the presence of two or more chronic communicable OR non-communicable diseases within an individual.^1^ Having a chronic non-communicable diseases and at least one communicable disease and vice versa was considered as having communicable and non-communicable disease multimorbidity. Multimorbidity was further classified as occurring either concordantly (the presence of two or more related chronic conditions with underlying similar etiology, for example, diabetes and hypertension) or discordantly (the presence of two or more chronic conditions, for example, tuberculosis and type 2 diabetes, with differences in etiology and management).^1^

We described communicable diseases as infectious diseases, whose direct or indirect spread from one individual to another, is either through contact with contaminated surfaces, bites from disease vectors, and ingestion of infested food or water.^17^ This was also including infections from bacteria and viruses which are carried in the mouth, nose, throat, and respiratory tract and those infections that are spread following exposure to bodily fluids such as blood and secretions. Examples of these diseases are HIV, TB, peptic ulcer disease (PUD), and viral hepatitis A, B, and C.^17^ Chronic non-communicable diseases were defined as non-infectious diseases with gradual onset and progress.^18^ Examples of such diseases are CVDs (congestive heart failure, ischemic heart disease, hypertension, and high blood pressure), type 2 diabetes, chronic lung diseases (chronic bronchitis, chronic obstructive pulmonary disease, and asthma), musculoskeletal diseases, cancer, thyroid diseases, depression, anxiety, and visual and hearing problems.^18^

Multimorbidity patterns were defined based on the frequency of the occurrence of a combination of two or more chronic communicable diseases and non-communicable diseases or the extent of the association of particular groups of disease conditions based on study sample group characteristics (cluster and factor analysis).^1^ Multimorbidity pattern in this review should compose of at least one communicable and one non-communicable disease. Determinants were defined at the societal level and included the social, structural, and behavioral characteristics of the study population that influence the occurrence of health-related events.^1^

### Study selection and eligibility of studies

Initial study selection was conducted independently by two reviewers (LK and OA). The process of selection of studies was conducted through database searches and backward citation checking. Studies reporting on original research either on prevalence, patterns, determinants, and management of multimorbidity of communicable diseases and non-communicable diseases in LMICs were identified. Following pre-determined eligibility criteria, two investigators (LK and OA) independently conducted the assessment for eligibility of studies. A third member of the review team (DB) was consulted in case of discordance on the choice of included abstracts. All articles that met the inclusion criteria were retained for full text screening.

### Inclusion and exclusion criteria

Only articles where the original research reported on a combination of communicable and non-communicable diseases were included. All articles reporting only on communicable diseases or non-communicable diseases, a single non-communicable diseases or communicable diseases, chronic disease co-morbidity/co-occurrence, systematic reviews, and single case studies were excluded. The differentiation between studies reporting on multimorbidity and those reporting on chronic co-morbidity/co-occurrence was made based on whether the participant had an underlying index disease/condition. Included multimorbidity studies reported on the co-occurrence of two or more chronic conditions within an individual. Studies reporting on chronic co-morbidity/co-occurrence had a focus on an underlying index condition, therefore they were excluded. We focused on studies conducted in LMIC based on the 2019 World Bank country classification on income level,^19^ involving adult subjects aged 18 years or over from any study settings such as the general population, resident households, patients in tertiary hospitals, primary care health facilities, and home-based care institutions.

Ineligibility of studies was assessed based on the characteristics of the study population, language of full text article, and primary focus of study. Studies published in a language other than English, reporting only on communicable diseases or non-communicable diseases, a single non-communicable disease or communicable disease, chronic disease co-morbidity/co-occurrence, and single case studies were excluded. Evidence shows that restricting systematic reviews to English-language publications appears to have little impact on the effect estimates and conclusions of systematic reviews.^20^

### Data extraction, synthesis, and analysis

Information on i) study characteristics—name of the author, year of publication, study design, data source, sample size and sample characteristics, socio-demographic characteristics such as age range of study subjects, number of chronic conditions, and information on data handling (how studies identified missingness patterns and the management of missing data); ii) factors associated with multimorbidity; iii) prevalence of multimorbidity expressed in percentage; and iv) patterns of multimorbidity were extracted from full text articles.

Findings from the included studies were presented in a narrative format. The following summary measures were included: Odds ratios (OR) for the association between risk factors/determinants and communicable and non-communicable disease multimorbidity, and disease prevalence.

### Quality assessment of studies

The National Institute of Health (NIH) quality assessment tool for Observational Cohort and Cross-Sectional Studies was used to critically appraise the reliability, validity, and overall quality of included articles.^21^ Two investigators (LK and OA) applied the assessment criteria to rate the quality of the included studies independently and in parallel. Any discrepancies in their findings were discussed and resolved. The standard assessment was done with reference to: i) Appropriateness of the research objective to the research question of the systematic review; ii) the study population and eligibility of participation; iii) provision of sample size justification; iv) study power; v) variable measurements of exposure and outcome; vi) clear definition of dependent and outcome variables; vii) report on loss to follow-up; viii) blinding of participant or investigator; ix) identification of covariates; and x) stratification and descriptive analyses. Articles were either rated as good, fair, or poor. An explanation was provided by each reviewer following the quality rating on every article. Any misclassifications or rating disagreements were resolved in consultation with a third investigator (DB) and with the research team where necessary.

## Results

**Figure 1.**
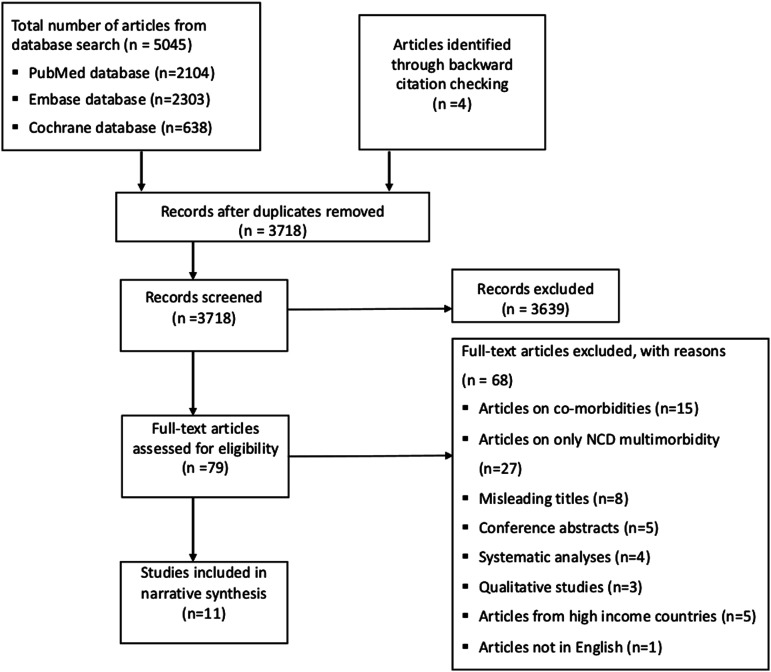
Flow diagram illustrating the article selection process.

Following database searches and backwards citation checking, a total of 3718 articles were available for screening after duplicate removal. Seventy-nine articles were relevant and their full texts were reviewed. Out of this, a total of 68 articles were excluded. This included articles reporting only non-communicable disease multimorbidity (n = 27), co-morbidities (n = 16), articles with a misleading multimorbidity title (where the title featured the term “multimorbidity” but the research focus was not on multimorbidity) (n = 8), systematic reviews (n = 4), and single case studies (n = 2). This resulted in eleven included articles in a narrative and quantitative synthesis (Figure 1).

### Study population, study design, and sample characteristics

Nine of the eleven included articles (n = 9) were single country studies,^6,22–29^ while the remaining two studies (n = 2) were multinational studies undertaken in 46 and 48 LMICs.^5,30^ Majority of the single country studies (n = 5) were conducted in South Africa. The study design applied across all studies was cross-sectional. Facility/institutional data was the most frequent source of data^24,25,27–29^ with some studies utilizing data from the World Health Organization (WHO) World Health Surveys^5,30^ and regional routine electronic pharmacy and chronic disease dispensing databases.^6^ Most studies applied probability sampling, with the exception of four studies. Three out of the four studies used non-probability sampling,^25,27,28^ while the remaining one study^24^ utilized both probability and non-probability sampling methods. The overall number of respondents from all included studies was 374,409 and sample sizes ranged from 491^25^ to 242,952^5^ from studies conducted in South Africa and 48 LMICs, respectively. All included studies included both males and females.

To establish presence of communicable diseases and non-communicable diseases, medical records,^25,27,29^ medication usage,^29^ self-reported diagnosis,^5,6,22.24,26,29,30^ administered prescriptions,^28^ and anthropometric measurements^22^ were employed. The number of chronic conditions included ranged from 4^6^ to 20.^29^ The definition of multimorbidity as the co-occurrence of two or more chronic non-communicable diseases and at least one communicable disease and vice versa within an individual was similar across all studies. This was with the exception of two studies; one study^22^ that also provided a second multimorbidity definition based on groups of conditions (cardiometabolic disorders, HIV, and mental conditions) and another study^28^ where the definition was deduced based from the study results where the prevalence of multimorbidity was given

### Quality of included studies

Study quality was evaluated regarding sources of bias as well as the reporting and handling of missing data (Table 1). Following this evaluation, studies were either categorized as good, fair, or poor quality. Overall, all included articles were concluded to be of good quality. Three articles^21,24,26^ failed to report the handling of missing data. Details of the quality assessment of the included studies are presented as Supplementary file 3.

**Table 1.** Characteristics of included studies for multimorbidity communicable and non-communicable disease multimorbidity in LMICs.

Author year, country	Study design	Data sources	Sampling methods	Sample sizes	Age range (years) and gender female %	Number of chronic conditions	Prevalence of multimorbidity	Study quality
Pati et al. (2020), India^29^	Cross-sectional	Primary and secondary facility-based data	Probability	1649	≥18	20	28.3%	Good
					44.2%			
^a^Chang et al. (2019), South Africa^22^	Cross-sectional	Longitudinal study (HAALSI program)	Probability	3889	≥40	10	69.4%	Good
					55.0%			
^a^Chang et al. (2019), South Africa^23^	Cross-sectional	Longitudinal study (HAALSI program)	Probability	4447	≥40	10	Based on disease clusters	Good
					61.8%			
Vancampfort et al. (2017), 46 LMICS^30^	Cross-sectional	World Health Survey	Probability	228,024	≥18	9	13%	Good
					50.8%			
Heerden et al. (2017), South Africa^28^	Cross-sectional	Community-based data	Probability	570	≥18	5	56.0%	Good
					69.0%			
Pati et al. (2017), India^24^	Cross-sectional	Primary and secondary facility data	Probability and non-probability	1670	≥18	17	28.3%	Good
					55.8%			
Roche et al. (2017), South Africa^25^	Cross-sectional	Facility based data (Hospital)	Non-probability	491	≥18	12	87.0%	Good
					57.4%			
Ahmadi et al. (2016), Iran^26^	Cross-sectional	Golestan cohort study	Probability	50,045	≥40	8	19.4%	Good
					57.6%			
Fernandez et al. (2016), Indonesia^27^	Cross-sectional	Secondary facility-based data	Non-probability	22,550	≥18	10	Based on disease clusters	Good
					4.0%			
Stubbs et al. (2016), 48 LMICS^5^	Cross-sectional	World Health Survey	Probability	242,952	≥18	9	13.0%	Good
					50.6%			
Oni et al. (2015), South Africa^6^	Cross-sectional	Regional routine electronic pharmacy and chronic disease dispensing database	Probability	14,364	≥18	4	22.6%	Good
					71.0%			

^a^Same author, same year of publication, and different studies;

### Prevalence of multimorbidity

The prevalence of communicable and non-communicable disease multimorbidity was assessed in all studies. The prevalence of multimorbidity increased with age, with a high prevalence (>30.0%) also noted among those aged 40 years and below.^5,22^ The prevalence varied from 13.0%^5^ in a multinational study conducted in 48 LMICs with 242,952 participants to 87% in one study conducted in South Africa with 411 participants.^25^ In some studies, the prevalence was reported based on disease combinations.^23,27^ All reported prevalence across the included studies surpassed 20%, except in two studies; a multinational study conducted in 48 LMICs^5^ and another study conducted in Iran^26^ with prevalence of 13.0 % and 19.4%, respectively) (Table 1).

### Factors associated with multimorbidity

All studies reported a higher prevalence of multimorbidity among female participants compared to their male counterparts. In one study,^5^ the multimorbidity prevalence of females was nearly double that of the male participants (Table 1 and Supplementary Figure S1 and S2). The highest multimorbidity prevalence was reported among the older population >50 years of age,^5,23,24,26,29^ the unemployed,^26^ and women^22,24,29^ except for one study. With regards to wealth quintiles, the prevalence of multimorbidity was found to be higher among those from the least wealth quintile with the exception of two studies^22,26^ that reported equal prevalence in both the most and least well-off, and a higher prevalence among the wealthiest quintile, respectively.

Age was the most frequently reported factor associated with multimorbidity (Tables 2 and 3). Higher odds of multimorbidity were significantly reported among older age groups >50 years,^5,22,26,30^ female participants,^5,26^ those with low physical activity^24,30^ and those currently working/employed^26^ as compared to those in younger age groups, male participants, with high physical activity and unemployed, respectively. Significantly lower odds of multimorbidity were also reported among people with university education/at tertiary level compared to those who were uneducated/with no formal schooling.^5,26^

**Table 2.** Associated factors of communicable and non-communicable disease multimorbidity.

Author (year)	Country	Age	Sex	Education	Wealth/income/SES	Working status	Residence (rural/urban)	Body mass index	Physical activity
Vancampfort et al. (2017)^30^	46 LMICS	X	X	X	X			X	
Stubbs et al. (2016)^5^	48 LMICS	X	X	X					
^a^Chang et al. (2019)^22^	South Africa	X	X	X	X	X			
^a^Chang et al. (2019)^23^	South Africa	X	X	X	X	X	X		
Pati et al. (2017)^24^	India	X	X	X	X		X		
Roche et al. (2017)^25^	South Africa	X	X						X
Ahmadi et al. (2016)^26^	Iran	X	X	X				X	X
Fernandez et al. (2016)^27^	Indonesia	X	X						
Oni et al. (2015)^6^	South Africa	X	X						
Heerden et al. (2017)^28^	South Africa	X		X	X			X	X
Pati et al. (2020)^29^	India	X	X	X					

^a^Same author, same year of publication, and different studies.

**Table 3.** Predictors of communicable and non-communicable disease multimorbidity.

Author (year)	Main category	Reference category	Odds ratio and 95% CI	Sample sizes
Age				
Ahmadi et al. (2016)^26^	61+	<=49	2.56 (2.39–2.75)	50,045
	50–60	<=49	1.69 (1.60–1.79)	50,045
Vancampfort et al. (2017)^30^	≤50	50–64	1.38 (1.19–1.6)	44,089
Stubbs et al. (2016)^5^	≤18	18–44	1.07 (1.06–1.07)	242,952
^a^Chang et al. (2019)^22^	60–69	40–49	1.07 (1.01–1.12)	3889
	80+	40–49	1.09 (1.02–1.16)	3889
Sex				
Stubbs et al. (2016)^5^	Female	Male	1.66 (1.56–1.77)	242,952
Ahmadi et al. (2016)^26^	Female	Male	2.11 (1.96–2.27)	50,045
Marital status				
*Chang et al. (2019)^22^	Separated/divorced	Currently married/living with a partner	1.07 (1.02–1.12)	3889
	Widowed	Currently married/living with a partner	1.09 (1.04–1.13)	3889
Income/wealth				
*Chang et al. (2019)^22^	Rich (Q4)^b^	Poorest (Q1)	1.06 (1.01–1.11)	3889
Ahmadi et al. (2016)^26^	Low SES	High SES	1.12 (1.04–1.21)	50,045
Working status				
Ahmadi et al. (2016)^26^	Currently not working	Currently working	1.46 (1.37–1.56)	50,045
Physical activity				
Vancampfort et al. (2017)^30^	Low	Vigorous	1.31 (1.21–1.42)	44,089
Ahmadi et al. (2016)^26^	No	Yes	1.21 (1.14–1.29)	50,045
Alcohol use				
Ahmadi et al. (2016)^26^	Ever	Never	1.44 (1.25–1.66)	50,045
Substance use				
Ahmadi et al. (2016)^26^	Ever	Never	1.73 (1.61–1.86)	50,045
Body mass index				
Ahmadi et al. (2016)^26^	Obese	Normal	2.33 (2.19–2.48)	50,045
	Overweight	Normal	1.62 (1.53, 1.72)	

^a^Same author, same year of publication, and different studies; SES = socioeconomic status; Q = quintile;

^b^Highest quintile is Q5.

Three studies examined the association between wealth quintile and multimorbidity^5,22,26^; two studies which classified wealth based on Q1 (poorest) to Q5 (richest)^22^ and based on low, medium, and high socioeconomic status^26^ found significantly higher odds of multimorbidity among the groups classified as Q4 (OR 1.06; 95% CI 1.01–1.11) and low (OR 1.12; 95% CI 1.04–1.21) as compared to Q1 and high socioeconomic status, respectively. A study conducted in Iran, with 50,045 participants, reported higher odds of multimorbidity for obese participants (OR 2.33; 95% CI 2.19–2.48) and those who were overweight (OR 1.62; 95% CI 1.53–1.72) as compared to those with normal weight.^26^ The same study also found a significantly positive association between multimorbidity and the consumption alcohol (OR 1.44; 95% CI 1.25–1.66).^26^ Only one multinational study conducted in 48 LMICs, with 242,952 participants, found a higher odds of multimorbidity for those who were widowed (OR 1.07; 95% CI 1.02–1.12) and those recently separated/divorced (OR 1.09; 95% CI 1.04–1.13) compared with those who were currently married/living with a partner.^5^

### Patterns of multimorbidity

**Figure 2.**
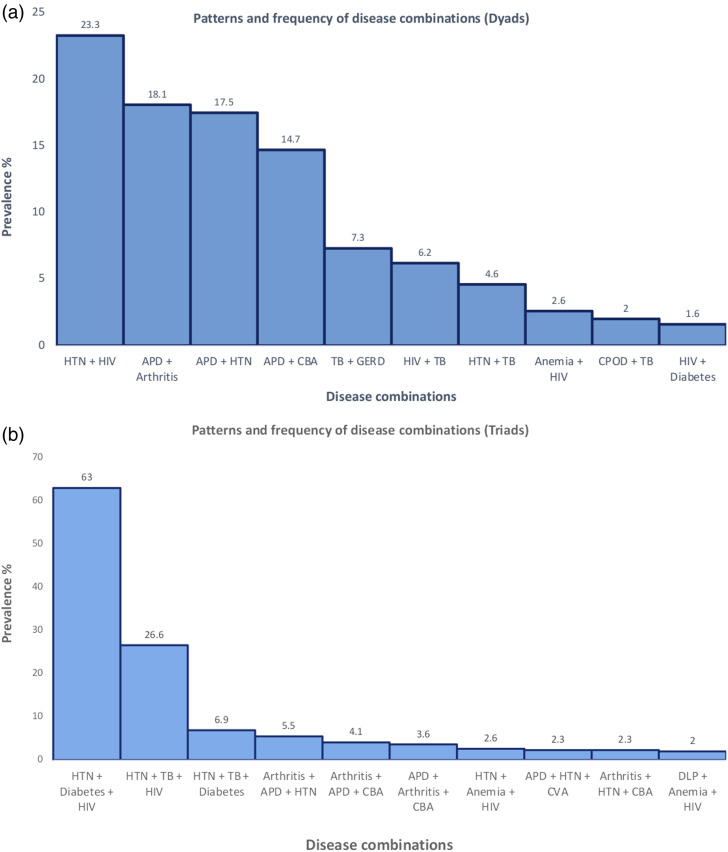
Patterns of CD and NCD multimorbidity (dyads).

Five articles reporting on individual country studies^6,22,24,27,29^ and one article reporting on 48 LMIC studies^5^ provided information on the patterns of multimorbidity (Table 4, Figures 2(a) and (b)). Combinations of two concurrent communicable and non-communicable diseases,^6,22,24,27^ three concurrent communicable and non-communicable diseases,^6,22,24^ and sex-specific communicable and non-communicable disease combinations^24^ were observed. One study analyzed both dyads and triads.^24^ The most frequently occurring dyads were HIV and hypertension (23.3%), and acid peptic disease and hypertension (18.2 %). HIV, hypertension, and diabetes were the most frequently occurring triad (63.0 %), followed by HIV, TB, and diabetes (26.6%).^5,6,22,24,27,29^

**Table 4.** Patterns of communicable and non-communicable disease multimorbidity in LMICs.

Author (year)	Statistical method	Multimorbidity patterns
*Chang et al. (2019)^22^	Descriptive analysis	Anemia + HIV (2.6%)
		Hypertension + HIV (1.9%)
		Hypertension +anemia + HIV (2.6%)
		Dyslipidemia + anemia + HIV (2.0%)
Ahmadi et al. (2016)^26^	Descriptive analysis	Tuberculosis + gastro-esophageal reflux disease (7.3%)
		Cardiovascular disease + tuberculosis (4.6%)
		Chronic obstructive pulmonary disease + tuberculosis (1.9%)
Pati et al. (2017)^24^	Descriptive analysis	Men group (dyad)
		Acid peptic disease + arthritis (7.9%)
		Acid peptic disease + hypertension (7.0%)
		Acid peptic disease + chronic backache (6.6%)
		Women group (dyad)
		Acid peptic disease + hypertension (10.5%)
		Acid peptic disease + arthritis (10.2%)
		Acid peptic disease + chronic backache (8.1%)
		Men group (triad)
		Acid peptic disease + arthritis + chronic backache (3.6%)
		Arthritis + acid peptic disease + hypertension (2.4%)
		Acid peptic disease + hypertension + chronic backache (2.3%)
		Women group (triad)
		Arthritis + acid peptic disease + chronic backache (4.1%)
		Arthritis + acid peptic disease + hypertension (3.1%)
		Arthritis + hypertension + chronic backache (2.3%)
Oni et al. (2015)^6^	Descriptive analyses	Double morbidities
		Hypertension + HIV (21.4%)
		HIV + tuberculosis (6.2%)
		HIV + type 2 diabetes mellitus (1.6%)
		Triple morbidities
		Hypertension + type 2 diabetes mellitus + HIV (63.0%)
		Hypertension + tuberculosis + HIV (26.6%)
		Hypertension + tuberculosis + type 2 diabetes mellitus (6.9%)

### Health service utilization

Two articles reported on the management of multimorbidity of communicable and non-communicable diseases^23,29^ including utilization of healthcare services at private and public health facilities,^29^ medication use,^29^ and continuum of care.^23^ A South African study,^23^ with participants 40 years and older, reported that patients with concordant multimorbidity (e.g., diabetes/hypertension patients with cardiometabolic conditions) had better diagnostic rates and continuum of care compared to those with discordant multimorbidity (e.g., HIV patients with cardiometabolic conditions). Patients with concordant multimorbidity reportedly had timely detection and diagnosis of chronic conditions (first stages in the care continuum) compared to their counterparts with discordant multimorbidity. Patients with discordant multimorbidity comparatively reported more challenges when starting a treatment course and adhering to the treatment regimen (last stages in the care continuum).^23^

A study from India,^29^ with participants 18 years and older, showed that the odds of visiting public health facilities was highest among participants >70 years (OR 26.29; 95% CI 10.52–65.66) and female multimorbidity patients (OR 1.6; 95% CI 1.1–1.3). The highest odds of visiting private health facilities were reported among those from high income level (OR 1.35; 95% CI 1.01–2.06). In addition, the highest percentage of patients attending private health facilities were those with single morbidities (n = 470) (28.5%) compared to those with multimorbidity (n = 208) (12.6%). Regarding the use of medication for multimorbidity, females above 40 years and males above 50 years had the highest medication use. Multimorbidity patients (females aged over 40 years and males aged over 50 years) using public health facilities reported twice the average number of medications compared to other age groups. However, both female and male multimorbidity patients using private health facilities reported a decrease in the number of medications taken after the age of 60 years.^29^

## Discussion

### Summary of main findings

This systematic review comprehensively describes the prevalence, patterns, determinants, and healthcare challenges of communicable and non-communicable disease multimorbidity in 374,409 participants. The reported prevalence of multimorbidity from the included studies varied from 13% to 87%. The odds of multimorbidity were higher among participants older than 50 years of age, female participants, the least educated/with no formal schooling, physically inactive, and obese participants. Disease patterns/ combinations were based on frequently occurring communicable diseases (HIV, tuberculosis, and peptic ulcer disease) and non-communicable diseases (CVDs and type 2 diabetes). Higher healthcare costs/expenditure were noted for patients with communicable and non-communicable disease multimorbidity compared to those with single morbidities.

### Discussion of main findings

This systematic review found a prevalence of communicable and non-communicable disease multimorbidity between 13% and 87%, with substantial variation across studies. Similar variations in the prevalence of multimorbidity have also been observed in reviews that looked at multimorbidity of NCDs in LMICs,^15,31^ South Asia,^32^ and HICs.^33,34^ While the primary and secondary healthcare facilities in most LMICs have vertical programs designed to handle the management of single chronic conditions, the emerging burden of communicable and non-communicable disease multimorbidity has added to an already present challenge in multimorbidity management.^1,3^

Variations in prevalence of communicable and non-communicable disease multimorbidity in different settings could be attributed to differences in the operational definition of multimorbidity used in the included studies, the study sample, sampling methods, study settings, and sample size selection. The potential for over-estimation and under-estimation of the multimorbidity prevalence is high owing to different methods of conducting the research, different choices of study settings, and instruments used by the different studies. For instance, the highest prevalence of communicable and non-communicable disease multimorbidity (87%) was reported in studies conducted in hospital settings,^25^ with lower sample sizes^25,28^ and vice versa.^5,25^

The most frequently occurring dyads were HIV and hypertension (23.3%), and acid peptic disease and hypertension (18.2%). HIV, hypertension, and diabetes (63.0%) and HIV, TB, and hypertension (26.6%) were the most commonly occurring triads. Only one study provided a sex-specific pattern analysis.^24^ Female participants were reported to have a higher frequency of dyads (62.0 %) and triads (22.2%) compared to their male counterparts (48.8% and 15.7%), respectively. More studies should report on aggregated multimorbidity pattern analysis to guide targeted interventions for different sub-groups. Most studies in the systematic review ascertained presence of chronic conditions through self-reported diagnosis. There are challenges with the reliability, recall bias, and measurement bias of chronic conditions arising from self-reporting. Therefore, the chance of underreporting of targeted chronic conditions with self-report is high.^13^ Countries with high communicable disease endemicity are inclined to focus on endemic diseases having a significant contribution to the disease burden. For example, most studies conducted in South Africa reported on TB and HIV as their selected chronic conditions.^6,22,23,25,28^ Therefore, it is possible that other chronic conditions that are less contributory to the disease burden are overlooked. Most management programs are hence designed to address these prioritized chronic conditions.

While this systematic review found the associations of risk factors with multimorbidity, causal interactions could not be established due to the cross-sectional nature of the included studies. Consistent with studies from HIC,^33,34^ the included studies reported a significantly positive association between age and multimorbidity. With the exception of a multinational study with a sample size of 242,952 that reported significantly higher odds of multimorbidity among participants 18–44 years of age compared to those 45 years and older, all other included studies in the review reported the highest odds of multimorbidity among participants older than 50 years. Comparative findings from LMICs reporting on chronic non-communicable disease multimorbidity^15,31^ have also found positive associations between multimorbidity and age. For instance, a scoping review investigating the multimorbidity of chronic non-communicable diseases in low- and middle-income countries found that included studies reported varied ranges in the prevalence of multimorbidity across different age groups—3% to 68%, 19% to 80%, and 27% to 91% for adults aged over 18 years, 40 years, and 60 years, respectively.^31^ Methodological differences including sample size selection could have impacted on result findings. With increase in age, the likelihood of developing other chronic conditions also increases.^1,3,35^ However, studies have also reported a rise in multimorbidity prevalence among the younger age groups.^5^ Therefore, it is imperative that communicable and non-communicable disease multimorbidity management interventions and policies target older adults but are also inclusive of younger adults.^35,36^

Female participants were observed to have significantly higher odds of communicable and non-communicable disease multimorbidity compared to their male counterparts. These findings are consistent with the 2018 Academy of Medical Sciences report on multimorbidity indicating that females experience two or three-fold the burden of multimorbidity as compared to males.^1^ Higher odds of multimorbidity in females have been attributed to a higher life expectancy,^37^ more self-reporting,^38^ and higher frequency visits to healthcare facilities.^39^ Females are more likely to attend routine screening for chronic conditions, hence have higher chances of early diagnosis compared to males^38,39^. Therefore, programs targeted toward behavior change to promote early screening of chronic diseases and frequent visits to primary healthcare facilities should be prioritized for both males and females in LMICs.

This systematic review reports significantly higher odds of communicable and non-communicable disease multimorbidity among both the most and the least well-off participants. In addition, the review also describes higher odds of multimorbidity among people with no education/formal training. However, there is limited evidence on the association of socioeconomic status (SES) with communicable and non-communicable disease multimorbidity to establish consistency with the systematic review findings. Most LMIC studies have focused on socioeconomic disparities in single disease conditions and in non-communicable disease multimorbidity/co-morbidity^40,41^ as opposed to communicable and non-communicable disease multimorbidity.^42^ Without sufficient evidence on the socioeconomic distribution of communicable and non-communicable disease multimorbidity within population groups in LMICs, establishing interventions directed at addressing these social determinants of health remains a difficult task. Knowledge on communicable and non-communicable disease combinations is a research priority for the identification of risk factors in certain disease patterns.^43,44^

This systematic review offers scarce evidence on healthcare utilization and healthcare costs in multimorbidity despite a rising burden of communicable and non-communicable disease multimorbidity. Patients with communicable and non-communicable disease multimorbidity from LMICs incur high healthcare costs from primary and secondary healthcare facilities.^45^ Prioritizing integrated management of communicable and non-communicable disease multimorbidity could facilitate better detection and improved diagnostic rates in primary care facilities, reduce out of pocket expenditure (OOPE), and improve quality of life through prompt access to medications and access to holistic multimorbidity care within the same facility.^42^

### Strengths and limitations

This review is among the first systematic reviews to report on the prevalence, patterns, determinants, and healthcare challenges of communicable and non-communicable disease multimorbidity in LMICs. Therefore, the results and implications of findings from the review provide a reference point for future communicable and non-communicable disease multimorbidity research, and a starting point into policy formulation for multimorbidity related care in LMICs. This review offers a baseline for future research into the interaction of communicable and non-communicable disease, multimorbidity patterns, and clustering.

A limitation of this systematic review is a significant heterogeneity across included studies. This is due to the lack of standardization in study methods and participant selections. Due to this high level of heterogeneity, we did not establish an overall effect estimate of the included studies. As in other systematic reviews, there is a possibility that some published and unpublished research papers were missed and have not been included in the review. However, we tried to mitigate this by developing a structured and systematic search strategy with the help of a University Liberian. Applying language restrictions (only articles in the English language) during article screening may have also limited our scope of research articles on communicable and non-communicable disease multimorbidity.

### Implications of research findings, research gap, and priority

Overall, there is a wide research gap in the context of non-communicable diseases combined with frequently occurring communicable diseases such as HIV and TB. To address the complex care needs of individuals living with communicable and non-communicable disease multimorbidity as well as improve access and delivery of care, better prevention and management strategies are needed. The concept of service integration either at full or partial capacity has been explored.^46–48^ Integration at different levels of the healthcare system can ensure improved access and prompt delivery of services. In endemic LMIC settings, the integration of non-communicable disease programs within already well-established communicable disease management programs will be beneficial.^46,47^ Individuals living with communicable and non-communicable disease multimorbidity will benefit from optimal multimorbidity care through combined programs and service delivery in addition to coordinated management decisions by the involved healthcare providers.^47^ However, there is limited evidence on the effectiveness of integrated care models applied in LMICs. Challenges including but not limited to poor structural guidelines on integration, poor governance in resource allocation, execution, and evaluation of integrated systems have been reported.^47,49^ More studies are needed to examine how integration can be best achieved in different LMIC settings. A most recent review examining the integration of care for TB and non-communicable disease in LMICs recommends tackling communicable and non-communicable disease multimorbidity within the same facility and has prompted policy makers to consider the barriers and enablers to program implementation and evaluation. Also, policy makers should address underlying factors impacting on the healthcare system which is pertinent to the sustainability and effectiveness of programs.^50^

There is also a need to further explore the interaction of communicable diseases and non-communicable diseases within different sub-populations. Identification of disease clusters and patterns, and the effects of communicable and non-communicable disease multimorbidity on different sub-groups, will ensure that management guidelines are specifically directed toward the most affected sub-groups. Also, identifying risk factors among people within the same clusters will ensure targeted prevention interventions and easier mapping of communicable and non-communicable disease multimorbidity.

To identify possible causal factors and relationships longitudinal studies should be prioritized. This is critical for future planning of prevention programs and formulation of specific guidelines based on known multimorbidity patterns and trends. Inconsistencies in LMIC study findings, due to variations in study methodologies, designs, and data presentation, have affected the reproducibility and comparability of results. To ensure proper evidence-based recommendations for communicable and non-communicable disease multimorbidity, future research should focus on identifying an operational definition of multimorbidity and a standard list of definitions for chronic diseases (communicable or non-communicable). Also, future research should strive to develop a standard classification of multimorbidity and a select list of which chronic diseases need to be considered when measuring multimorbidity.^51^

## Conclusion

This systematic review reports a high prevalence of communicable and non-communicable disease multimorbidity among both the younger and older population groups, with variations in associations between multimorbidity and different determinants, and variations in communicable and non-communicable disease multimorbidity patterns. To meet the complex healthcare needs and facilitate better detection, diagnosis, and adherence to treatment for communicable and non-communicable disease multimorbidity patients, policy makers need to address underlying challenges and enablers in the healthcare system. This will promote better multimorbidity care and alleviate the psychosocial and financial burden experienced by patients. Identification and prevention of risk factors and addressing xevidence gaps in multimorbidity clustering is crucial to tackling the increasing communicable and non-communicable disease multimorbidity in LMICs and when planning for targeted interventions.

## Supplemental Material

Supplemental Material - Multimorbidity of communicable and non-communicable diseases in low- and middle-income countries: A systematic review Click here for additional data file. Supplemental Material for Multimorbidity of communicable and non-communicable diseases in low- and middle-income countries: A systematic review by Lucy Kaluvu, Asogwa Ogechukwa, Anna Marza Florensa, Catherine Kyobutungi, Naomi S Levitt, Daniel Boateng, and Kerstin Klipstein-Grobusch in Journal of Multimorbidity and Comorbidity
