# Supplementary material for: Multimorbidity of communicable and non-communicable diseases in low- and middle-income countries: A systematic review
Source: J Multimorb Comorb. 2022 Sep 1;12:26335565221112593. doi: 10.1177/26335565221112593 (PMC9445468; doi:10.1177/26335565221112593)
Supplement: Supplemental Material - Multimorbidity of communicable and non-communicable diseases in low- and middle-income countries: A systematic review [file sj-pdf-1-cob-10.1177_26335565221112593.pdf]

Author name, year, country of study

Vancampfort D(2017) 48 LMICS

Stubbs B (2016) 48 LMICS

Rodriguez-Fernandez R(2016) Indonesia

Roche S(2017) South Africa

Pati S(2017) India

Chang AY(2019) South Africa

Chang AY(2019) South Africa, Mozambique

Ahmadi B(2016) Iran

Pati S(2020) India

Heerden (2017) South Africa

Was the research question or objective in this paper clearly stated and appropriate	Was the study population clearly specified and defined
Yes	Yes
Yes	Yes
Yes	Yes
Yes	Yes
Yes	Yes
Yes	Yes
Yes	Yes
Yes	Yes
Yes	Yes
Yes	Yes

Was the participation rate of eligible persons at least 50%	Were all the subjects selected or recruited from the same or similar populations (including the same time period)?

yes	No
Yes	No
Yes	Yes
Yes	Yes
Yes	Yes
Yes	Yes
Yes	No
Yes	Yes
Yes	Yes
Yes	Yes

**Were inclusion and exclusion criteria for being in the study prespecified and applied uniformly to all participants?**	**Was a sample size justification, power description, or variance and effect estimates provided?**
Yes	NR
Yes	N/A
Yes	N/A
Yes	N/A

Yes	Yes
Yes	N/A
Yes	N/A
Yes	N/A
Yes	Yes
Yes	N/A

For the analyses in this paper, were the exposure(s) of interest measured prior to the outcome(s) being measured?	Was the timeframe sufficient so that one could reasonably expect to see an association between exposure and outcome if it existed?	For exposures that can vary in amount or level, did the study examine different levels of the exposure as related to the outcome (e.g., categories of exposure, or exposure measured as continuous variable)?
N/A	N/A	Yes
N/A	N/A	Yes
N/A	N/A	Yes
N/A	N/A	Yes
N/A	N/A	Yes
N/A	N/A	Yes
N/A	N/A	Yes
N/A	N/A	Yes

N/A	N/A	Yes
N/A	N/A	Yes

Were the exposure measures (independent variables) clearly defined, valid, reliable, and implemented consistently across all study participants	Was the exposure(s) assessed more than once over time?
Yes	N/A
Yes	N/A
Yes	N/A
Yes	N/A
Yes	N/A
Yes	N/A
Yes	N/A
Yes	N/A
Yes	N/A
Yes	N/A

Were the outcome measures (dependent variables) clearly defined, valid, reliable, and implemented consistently across all study participants?	Were the outcome assessors blinded to the exposure status of participants	Was loss to follow-up after baseline 20% or less?
Yes	No	N/A
Yes	No	N/A
Yes	No	N/A
Yes	No	N/A
Yes	No	N/A
Yes	No	N/A
Yes	No	N/A
Yes	No	N/A
Yes	No	N/A
Yes	No	N/A

Confounding variables measured and adjusted	Quality Rating
Yes	GOOD
Yes	GOOD
NR	GOOD
NR	GOOD
Yes	GOOD
Yes	GOOD
Yes	GOOD
Yes	GOOD
Yes	GOOD
Yes	GOOD

Comment

multimorbidity clearly defined, self-report plus symptom-based diagnosis, large sample size, adjusted for confounders

Multimorbidity is clearly defined, descriptive analyses was done to characterize the study sample, inclusion and exclusion criteria was clear, information on missing data provided, sample size large enough, power of study is high

Multimorbidity was defined-use of multimorbidity wheel, no information on missing data handeling, selection bias may have occurred due to convinient sampling (mine workers)

Multimorbidity was defined, selection bias may be present as the study excluded discharged and diseased patient's data as missing, adequate sample size and strong power of study

multimorbidity was clearly defined, self-report leads to chance of recall bias, inclusion and exclusion criteria clearly defined, sample size calculated and reported, missing data information missing

multimorbidity was clearly defined- two definitions given, missing data handeling was reported, exclusion and inclusion criteria clear, self-report

concordant and discordant multimorbidity was clearly defined, information on missing data was reported+ missing data handeling, self-report may have resorted in reporting bias

multimorbidity was well-defined, missing data reported and handeling information provided, self -report may have resulted in recall bias, confounders adjusted

Multimorbidity was well-defined, sample size calculations were provided,

outcome and dependent variables were defined, adjustments for age and gender were done, self-report, no information on missing data handling was provided

Multimorbidity was well-defined, outcome and dependent measures were clearly outlines and defined, there was no information on the handling of missing data, adjustment for age and HIV status was sone

Supplementary figures

Figure S1: Age-related prevalence of CD and NCD multimorbidity

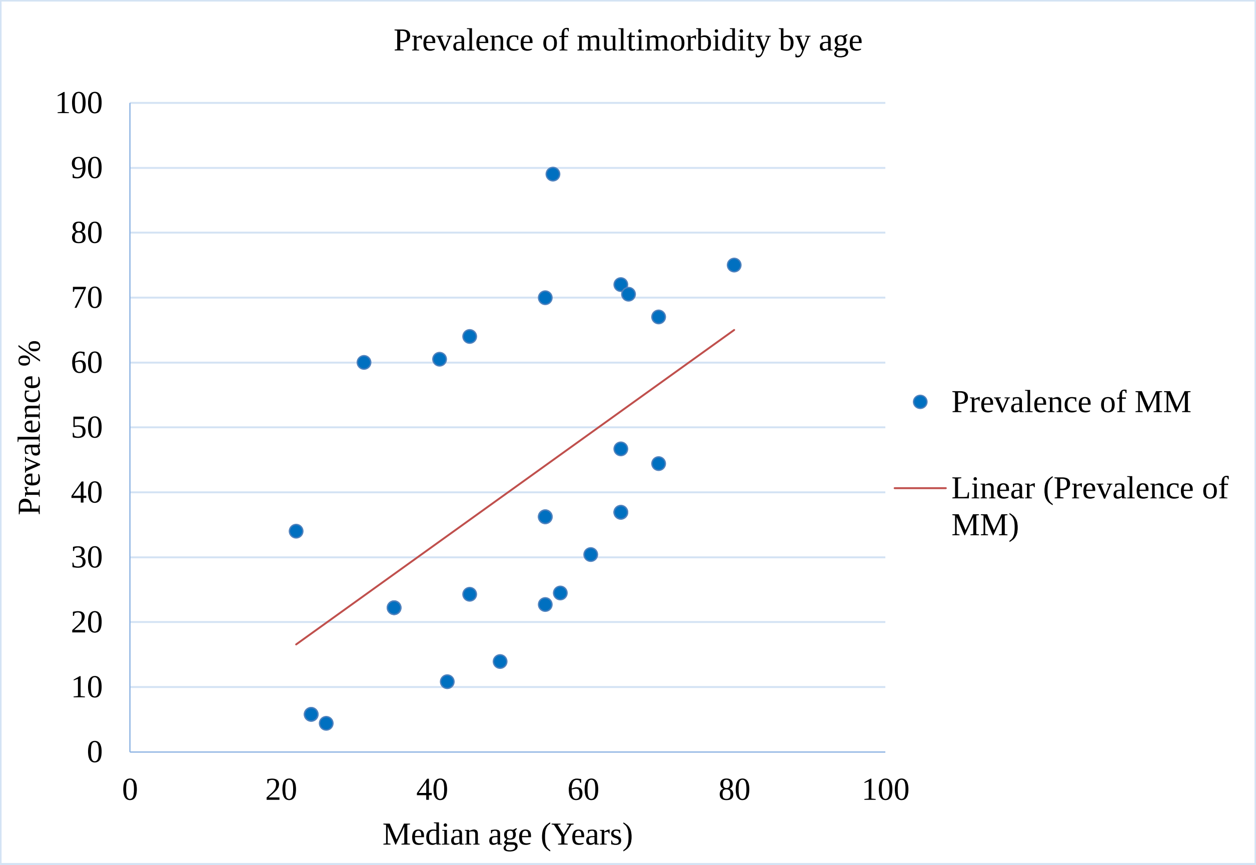

Figure S2: Sex-related prevalence of CD and NCD multimorbidity

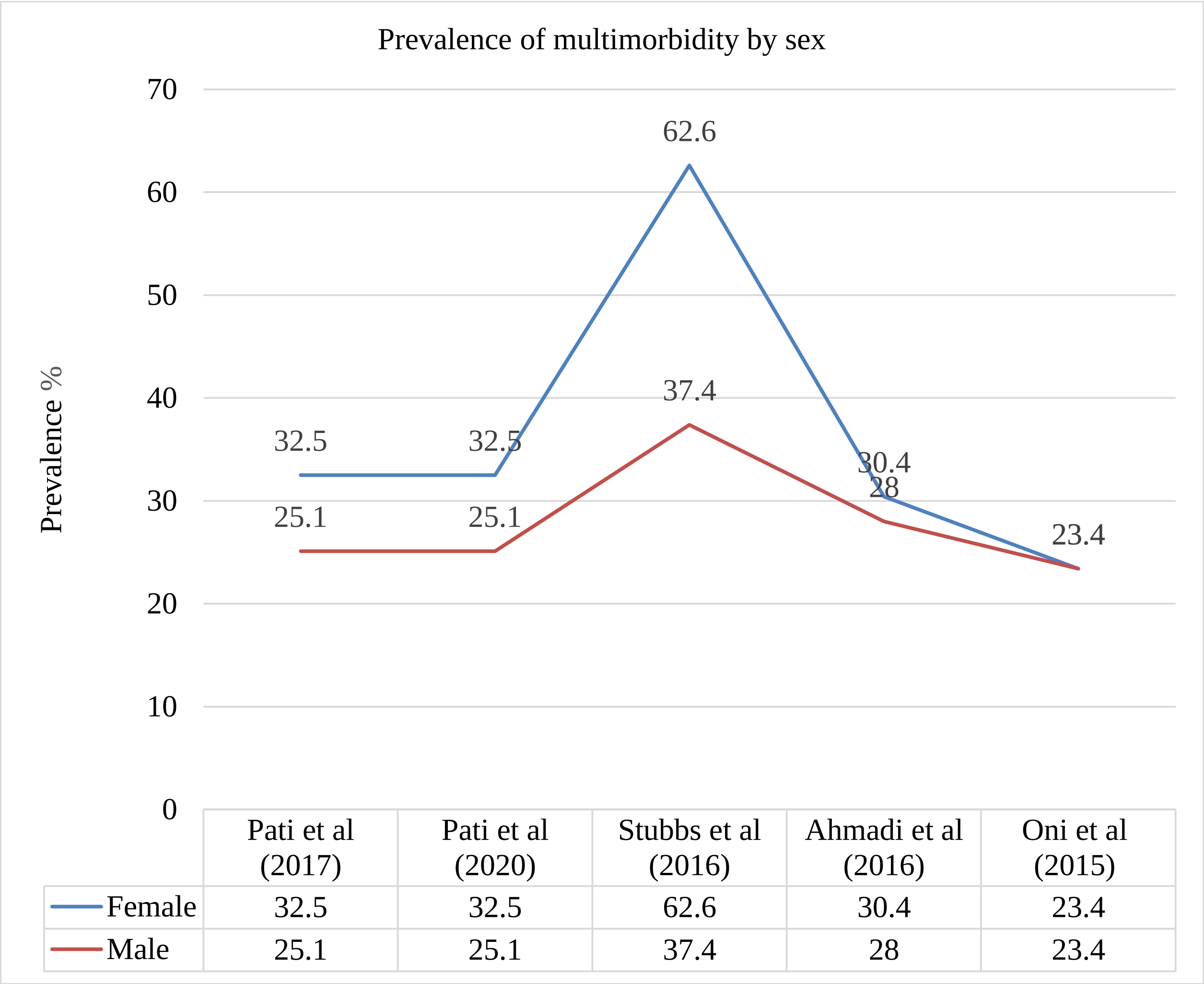

MULTIMORBIDITY OF COMMUNICABLE AND NONCOMMUNICABLE DISEASES IN LOW- AND MIDDLE-INCOME COUNTRIES: A SYSTEMATIC REVIEW AND META-ANALYSIS

Combined search strategy-PubMed, Embase and Cochrane

1. PUBMED

#9	Add	Search **((#3 AND #4 AND #7 AND #8))**	1917	07:17:06
#8	Add	Search **((Developing countries[MeSH terms]) OR developing countr*[Title/Abstract]) OR developing nation*[Title/Abstract]) OR developing population*[Title/Abstract]) OR developing econom*[Title/Abstract] OR undeveloped countr*[Title/Abstract] OR undeveloped nation*[Title/Abstract] OR undeveloped econom*[Title/Abstract] OR undeveloped econom*[Title/Abstract] OR least developed countr*[Title/Abstract] OR least developed nation*[Title/Abstract] OR least developed econom*[Title/Abstract] OR least developed econom*[Title/Abstract] OR less-developed countr*[Title/Abstract] OR less-developed nation*[Title/Abstract] OR less-developed population*[Title/Abstract] OR less-developed population*[Title/Abstract] OR less-developed econom*[Title/Abstract] OR lesser developed countr*[Title/Abstract] OR lesser developed nation*[Title/Abstract] OR lesser developed econom*[Title/Abstract] OR lesser developed population*[Title/Abstract] OR lesser developed population*[Title/Abstract] OR lesser developed econom*[Title/Abstract] OR under-developed countr*[Title/Abstract] OR under-developed nation*[Title/Abstract] OR underdeveloped countr*[Title/Abstract] OR underdeveloped nation*[Title/Abstract] OR underdeveloped population*[Title/Abstract] OR underdeveloped econom*[Title/Abstract] OR low income countr*[Title/Abstract] OR middle income countr*[Title/Abstract] OR low income nation*[Title/Abstract] OR middle income nation*[Title/Abstract] OR low income population*[Title/Abstract] OR middle income population*[Title/Abstract] OR low income econom*[Title/Abstract] OR middle income econom*[Title/Abstract] OR lower income countr*[Title/Abstract] OR lower income nation*[Title/Abstract] OR lower income population*[Title/Abstract] OR lower income economy*[Title/Abstract] OR lower income econom*[Title/Abstract] OR resource limited*[Title/Abstract] OR low resource countr*[Title/Abstract] OR lower resource countr*[Title/Abstract] OR low resource nation*[Title/Abstract] OR low resource population*[Title/Abstract] OR low resource econom*[Title/Abstract] OR low resource econom*[Title/Abstract] OR underserved countr*[Title/Abstract] OR underserved nation*[Title/Abstract] OR underserved population*[Title/Abstract] OR underserved econom*[Title/Abstract] OR under-served countr*[Title/Abstract] OR under-served countr*[Title/Abstract] OR under-served nation*[Title/Abstract] OR under-served population*[Title/Abstract] OR under-served population*[Title/Abstract] OR under-served econom*[Title/Abstract] OR under-served econom*[Title/Abstract] OR deprived countr*[Title/Abstract] OR deprived nation*[Title/Abstract] OR deprived economy*[Title/Abstract] OR deprived econom*[Title/Abstract] OR deprived population*[Title/Abstract] OR poor countr*[Title/Abstract] OR poor nation*[Title/Abstract] OR poor population*[Title/Abstract] OR poor econom*[Title/Abstract] OR poor countr*[Title/Abstract] OR poorer countr*[Title/Abstract] OR poorer nation*[Title/Abstract] OR poorer population*[Title/Abstract] OR poorer econom*[Title/Abstract] OR lmic*[Title/Abstract] OR lmics*[Title/Abstract] OR transitional countr*[Title/Abstract] OR transitional nation*[Title/Abstract] OR transitional econom*[Title/Abstract] OR low resource setting*[Title/Abstract] OR lower resource setting*[Title/Abstract] OR middle resource setting*[Title/Abstract] OR Third world*[Title/Abstract] OR South east asia*[Title/Abstract] OR Middle east*[Title/Abstract] OR Afghanistan*[Title/Abstract] OR Albania*[Title/Abstract] OR Algeria*[Title/Abstract] OR American samoa*[Title/Abstract] OR Angola*[Title/Abstract] OR Angolese*[Title/Abstract] OR Angolian*[Title/Abstract] OR Argentina*[Title/Abstract] OR Armenia*[Title/Abstract] OR Azerbaijan*[Title/Abstract] OR Bangladesh*[Title/Abstract] OR Belarus*[Title/Abstract] OR Belize*[Title/Abstract] OR Benin*[Title/Abstract] OR Bhutan*[Title/Abstract] OR Bolivia*[Title/Abstract] OR (Bosnia[Title/Abstract] AND Herzegovina*[Title/Abstract]) OR Botswana*[Title/Abstract] OR Brazil*[Title/Abstract] OR Bulgaria*[Title/Abstract] OR Burma*[Title/Abstract] OR Burkina Faso*[Title/Abstract] OR Burundi*[Title/Abstract] OR Cabo Verde*[Title/Abstract] OR Cambodia*[Title/Abstract] OR Cameroon*[Title/Abstract] OR Central Africa Republic*[Title/Abstract] OR Chad*[Title/Abstract] OR Comoros*[Title/Abstract] OR Congo*[Title/Abstract] OR Cote d'Ivore*[Title/Abstract] OR Cuba*[Title/Abstract] OR Djibouti*[Title/Abstract] OR Dominican Republic*[Title/Abstract] OR Ecuador*[Title/Abstract] OR East Africa*[Title/Abstract] OR Eastern Africa*[Title/Abstract] OR Egypt*[Title/Abstract] OR El Savador*[Title/Abstract] OR Equatorial Guinea*[Title/Abstract] OR Eritrea*[Title/Abstract] OR Eswatini*[Title/Abstract] OR Ethiopia*[Title/Abstract] OR Fiji*[Title/Abstract] OR Gabon*[Title/Abstract] OR The Gambia*[Title/Abstract] OR Georgia*[Title/Abstract] OR Ghana*[Title/Abstract] OR Grenada*[Title/Abstract] OR Guatemala*[Title/Abstract] OR Guinea*[Title/Abstract] OR Guinea Bissau*[Title/Abstract] OR Guyana*[Title/Abstract] OR Haiti*[Title/Abstract] OR India*[Title/Abstract] OR Indonesia*[Title/Abstract] OR Iran*[Title/Abstract] OR Iraq*[Title/Abstract] OR Jamaica*[Title/Abstract] OR Jordan*[Title/Abstract] OR Kazakhstan*[Title/Abstract] OR Kenya*[Title/Abstract] OR Kiribati*[Title/Abstract] OR Democratic People's Republic of Korea*[Title/Abstract] OR Kosovo*[Title/Abstract] OR Kyrgyz republic*[Title/Abstract] OR Lao**	1629404	07:16:04

		PDR[Title/Abstract] OR Lebanon*[Title/Abstract] OR Lesotho*[Title/Abstract] OR Liberia*[Title/Abstract] OR Libya*[Title/Abstract] OR Madagascar*[Title/Abstract] OR Malawi*[Title/Abstract] OR Malaysia*[Title/Abstract] OR Maldives*[Title/Abstract] OR Mali*[Title/Abstract] OR Marshall Islands*[Title/Abstract] OR Mauritania*[Title/Abstract] OR Mauritius*[Title/Abstract] OR Mexico*[Title/Abstract] OR Micronesia*[Title/Abstract] OR Moldova*[Title/Abstract] OR Mongolia*[Title/Abstract] OR Montenegro*[Title/Abstract] OR Morocco*[Title/Abstract] OR Mozambique*[Title/Abstract] OR Myanmar*[Title/Abstract] OR Namibia*[Title/Abstract] OR Nauru*[Title/Abstract] OR Nepal*[Title/Abstract] OR Nicaragua*[Title/Abstract] OR Niger*[Title/Abstract] OR Nigeria*[Title/Abstract] OR North Macedonia*[Title/Abstract] OR Pakistan*[Title/Abstract] OR Papua New Guinea*[Title/Abstract] OR Paraguay*[Title/Abstract] OR Peru*[Title/Abstract] OR Philippines*[Title/Abstract] OR Principe*[Title/Abstract] OR Romania*[Title/Abstract] OR Rhodesia*[Title/Abstract] OR Rwanda*[Title/Abstract] OR Samoa*[Title/Abstract] OR Sao tome*[Title/Abstract] OR Senegal*[Title/Abstract] OR Sierra Leone*[Title/Abstract] OR Solomon Islands*[Title/Abstract] OR Somalia*[Title/Abstract] OR South Africa*[Title/Abstract] OR Southern Africa*[Title/Abstract] OR South Sudan*[Title/Abstract] OR Sri Lanka*[Title/Abstract] OR Sudan*[Title/Abstract] OR Swaziland*[Title/Abstract] OR Syria*[Title/Abstract] OR Tajikistan*[Title/Abstract] OR Tanzania*[Title/Abstract] OR Togo*[Title/Abstract] OR Timor-Leste*[Title/Abstract] OR Togo*[Title/Abstract] OR Tonga*[Title/Abstract] OR Tunisia*[Title/Abstract] OR Tunis*[Title/Abstract] OR Uganda*[Title/Abstract] OR Uzbekistan*[Title/Abstract] OR Vanuatu*[Title/Abstract] OR Venezuela*[Title/Abstract] OR Vietnam*[Title/Abstract] OR (West Bank[Title/Abstract] AND Gaza*[Title/Abstract]) OR West Africa*[Title/Abstract] OR Yemen*[Title/Abstract] OR Zaire*[Title/Abstract] OR Zambia*[Title/Abstract] OR Zimbabwe*[Title/Abstract])))		
#7	Add	Search **((Non-communicable diseases[MeSH Terms]) OR Non-communicable diseases*[Title/Abstract]) OR Noncommunicable disease*[Title/Abstract]) OR Noninfectious disease*[Title/Abstract]) OR Non infectious disease*[Title/Abstract]) OR Non communicable chronic disease*[Title/Abstract]) OR Noncommunicable chronic disease*[Title/Abstract]) OR Cardiovascular diseases[MeSH Terms]) OR Cardiovascular disease*[Title/Abstract]) OR Heart disease*[Title/Abstract]) OR Heart failure*[Title/Abstract]) OR Coronary Infarction*[Title/Abstract]) OR Cardiac Infarction*[Title/Abstract]) OR Myocardial Infarction*[Title/Abstract]) OR Lung diseases, obstructive[MeSH Terms]) OR Chronic obstructive disease*[Title/Abstract]) OR Asthma*[Title/Abstract]) OR Emphysema*[Title/Abstract]) OR Chronic obstructive pulmonary disease*[Title/Abstract]) OR COPD*[Title/Abstract]) OR Cystic Fibrosis*[Title/Abstract]) OR Brochitis*[Title/Abstract]) OR Neoplasms[MeSH Terms]) OR Neoplasm*[Title/Abstract]) OR Cancer*[Title/Abstract]) OR Cancers*[Title/Abstract]) OR Tumor*[Title/Abstract]) OR Tumors*[Title/Abstract]) OR Tumour*[Title/Abstract]) OR Malignancies*[Title/Abstract]) OR Malignancy*[Title/Abstract]) OR Malignant*[Title/Abstract]) OR Malignant neoplasm*[Title/Abstract]) OR Malignant tumour*[Title/Abstract]) OR Diabetes mellitus[MeSH Terms]) OR Diabetes*[Title/Abstract]) OR Diabetic*[Title/Abstract]) OR Polygenic disease*[Title/Abstract]) OR Poligenic disorder*[Title/Abstract]) OR Mental disorders[MeSH Terms]) OR Mental disorders*[Title/Abstract]) OR Mental disorder*[Title/Abstract]) OR Depression*[Title/Abstract]) OR Depressive disorders*[Title/Abstract]) OR Personality disorders*[Title/Abstract]) OR Hypertension[MeSH Terms]) OR Hypertension[Title/Abstract]) OR Hypertensive disorders*[Title/Abstract]) OR Hypertensive*[Title/Abstract]) OR High blood pressure*[Title/Abstract])))**	8522951	07:15:41
#4	Add	Search **((Comorbidity[MeSH terms]) OR comorbidit*[Title/Abstract]) OR polymorbidit*[Title/Abstract]) OR multiple chronic[Title/Abstract]) OR multiple chronic medical conditions*[Title/Abstract] OR multiple morbidit*[Title/Abstract] OR multiple chronic health conditions*[Title/Abstract] OR multimorbidit*[Title/Abstract] OR multi-morbidit*[Title/Abstract])**	196144	07:01:00
#3	Add	Search **((Communicable Diseases[MeSH Terms]) OR communicable disease*[Title/Abstract]) OR communicable infection*[Title/Abstract] OR communicable chronic disease*[Title/Abstract] OR transmittable disease*[Title/Abstract] OR infectious disease*[Title/Abstract] OR transferrable disease*[Title/Abstract] OR contaminating disease*[Title/Abstract] OR virulent disease*[Title/Abstract] OR infective disease*[Title/Abstract] OR contagious disease*[Title/Abstract] OR HIV/AIDS[MeSH Terms] OR hiv*[Title/Abstract] OR aids*[Title/Abstract] OR wasting disease*[Title/Abstract] OR immunodeficiency virus*[Title/Abstract] OR immunodeficiency virus* OR wasting syndrome*[Title/Abstract] OR virulent disease*[Title/Abstract] OR transmissible disease*[Title/Abstract] OR transmissible infection*[Title/Abstract] OR Tuberculosis[MeSH Terms] OR tuberculos*[Title/Abstract] OR tuberculosis infection*[Title/Abstract] OR TB[Title/Abstract] OR T.B.[Title/Abstract] OR consumption*[Title/Abstract] OR white plague*[Title/Abstract] OR pott's disease*[Title/Abstract] OR bacilli infection*[Title/Abstract] OR pneumonia*[Title/Abstract] OR phthisis*[Title/Abstract] OR phthisic*[Title/Abstract] OR co-infection*[Title/Abstract] OR Malaria[MeSH Terms] OR malaria[Title/Abstract] OR plasmodium infection*[Title/Abstract] OR ectoparasitic infection*[Title/Abstract] OR ectoparasite*[Title/Abstract] OR Hepatitis[MeSH Terms] OR hepatitis[Title/Abstract] OR liver disease*[Title/Abstract] OR liver infection*[Title/Abstract] OR infectious disease*[Title/Abstract] OR hepatitis delta*[Title/Abstract] OR delta hepatitis*[Title/Abstract] OR hepatitis virus*[Title/Abstract] OR Hepatitis B[MeSH Terms] OR hepatitis b*[Title/Abstract] OR infectious hepatitis b*[Title/Abstract] OR serum hepatitis*[Title/Abstract] OR acute hepatitis b*[Title/Abstract] OR acute serum hepatitis*[Title/Abstract] OR Hepatitis C[MeSH Terms] OR hepatitis c*[Title/Abstract] OR Peptic Ulcer Disease[MeSH Terms] OR peptic ulcer disease*[Title/Abstract] OR peptic ulcer*[Title/Abstract] OR peptic ulceration*[Title/Abstract] OR active peptic ulceration*[Title/Abstract] OR gastric ulcer*[Title/Abstract] OR gastritis*[Title/Abstract] OR gastric infection*[Title/Abstract] OR gastric disease*[Title/Abstract]**	1701644	06:57:34

		OR duodenal ulcer*[Title/Abstract] OR duodenal infection*[Title/Abstract] OR duodenal disease*[Title/Abstract] OR ulcers*[Title/Abstract] OR pyloric infection*[Title/Abstract] OR pyloric disease*[Title/Abstract]))		

2. EMBASE

Embase Session Results (24 Feb 2020)

No.	Query	Results
#7	#6 AND [embase]/lim	**2173**
#6	#1 AND #2 AND #3 AND #4	**2383**
#4	'comorbidity'/exp OR cormobidit*:ab,ti OR polymorbidit*:ab,ti OR 'multiple chronic*':ab,ti OR 'multiple chronic medical condition*':ab,ti OR 'multiple morbidit*':ab,ti OR 'multiple chronic health condition*':ab,ti OR multimorbidit*:ab,ti OR 'multi morbidit*':ab,ti	**262018**
#3	('developing country'/exp/mj OR 'developing countr*':ab,ti OR 'developing nation*':ab,ti OR 'developing population*':ab,ti OR 'developing econom*':ab,ti OR 'undeveloped countr*':ab,ti OR 'undeveloped nation*':ab,ti OR 'undeveloped econom*':ab,ti OR 'least developed countr*':ab,ti OR 'least developed nation*':ab,ti OR 'least developed econom*':ab,ti OR 'less-developed countr*':ab,ti OR 'less-developed nation*':ab,ti OR 'less-developed population*':ab,ti OR 'less-developed econom*':ab,ti OR 'lesser developed countr*':ab,ti OR 'lesser developed nation*':ab,ti OR 'lessr developed econom*':ab,ti OR 'under-developed countr*':ab,ti OR 'under-developed econom*':ab,ti OR 'under-developed nation*':ab,ti OR 'underdeveloped countr*':ab,ti OR 'underdeveloped nation*':ab,ti OR 'underdeveloped population*':ab,ti OR 'underdeveloped econom*':ab,ti OR 'low income countr*':ab,ti OR 'middle income countr*':ab,ti OR 'low income nation*':ab,ti OR 'middle income nation*':ab,ti OR 'low income population*':ab,ti OR 'middle income population*':ab,ti OR 'low income econom*':ab,ti OR 'middle income econom*':ab,ti OR 'lower income countr*':ab,ti OR 'lower income nation*':ab,ti OR 'lower income population*':ab,ti OR 'lower income econom*':ab,ti OR 'resource limited*':ab,ti OR 'low resource countr*':ab,ti OR 'low resource nation*':ab,ti OR 'low resource population*':ab,ti OR 'low resource econom*':ab,ti OR 'underserved countr*':ab,ti OR 'underserved nation*':ab,ti OR 'underserved population*':ab,ti OR 'underserved econom*':ab,ti OR 'under-served countr*':ab,ti OR 'under-served nation*':ab,ti OR 'under-served populations*':ab,ti OR 'under-served econom*':ab,ti OR 'under-served economies':ab,ti OR 'deprived countr*':ab,ti OR 'deprived nation*':ab,ti OR	**1580542**

No.	Query	Results
	'deprived nations*':ab,ti OR 'deprived economies*':ab,ti OR 'deprived population*':ab,ti OR 'poor country*':ab,ti OR 'poor nation*':ab,ti OR 'poor population*':ab,ti OR 'poor economy*':ab,ti OR 'poor countries*':ab,ti OR 'poorer countr*':ab,ti OR 'poorer nation*':ab,ti OR 'poorer population*':ab,ti OR 'poorer econom*':ab,ti OR lmic*:ab,ti OR lmics*:ab,ti OR 'transitional countr*':ab,ti OR 'transitional nations*':ab,ti OR 'transitional economies*':ab,ti OR 'low resource setting*':ab,ti OR 'lower resource setting*':ab,ti OR 'middle resource setting*':ab,ti OR 'third world*':ab,ti OR 'south east asia*':ab,ti OR 'middle east*':ab,ti OR afghanistan*:ab,ti OR albania*:ab,ti OR algeria*:ab,ti OR 'american samoa*':ab,ti OR angola*:ab,ti OR angolese*:ab,ti OR angolian*:ab,ti OR argentina*:ab,ti OR armenia*:ab,ti OR azerbaijan*:ab,ti OR bangladesh*:ab,ti OR belarus*:ab,ti OR belize*:ab,ti OR benin*:ab,ti OR bhutan*:ab,ti OR bolivia*:ab,ti OR bosnia*:ab,ti OR herzegovina*:ab,ti OR botswana*:ab,ti OR brazil*:ab,ti OR bulgaria*:ab,ti OR burma*:ab,ti OR 'burkina faso*':ab,ti OR burundi*:ab,ti OR 'cabo verde*':ab,ti OR cambodia*:ab,ti OR cameroon*:ab,ti OR 'central africa republic*':ab,ti OR chad*:ab,ti OR comoros*:ab,ti OR congo*:ab,ti OR 'cote d`ivoire*':ab,ti OR cuba*:ab,ti OR djibouti*:ab,ti OR 'dominican republic*':ab,ti OR eucador*:ab,ti OR 'east africa*':ab,ti OR 'eastern africa*':ab,ti OR egypt*:ab,ti OR 'el savador*':ab,ti OR 'equatorial guinea*':ab,ti OR eritrea*:ab,ti OR eswatini*:ab,ti OR ethiopia*:ab,ti OR fiji*:ab,ti OR gabon*:ab,ti OR 'the gambia*':ab,ti OR georgia*:ab,ti OR ghana*:ab,ti OR grenada*:ab,ti OR guatemala*:ab,ti OR guinea*:ab,ti OR 'guinnea bissau*':ab,ti OR guyana*:ab,ti OR haiti*:ab,ti OR india*:ab,ti OR indonesia*:ab,ti OR iran*:ab,ti OR iraq*:ab,ti OR jamaica*:ab,ti OR jordan*:ab,ti OR kazakhstan*:ab,ti OR kenya*:ab,ti OR kiribati*:ab,ti OR 'democratic peoples republic of korea*':ab,ti OR kosovo*:ab,ti OR 'kyrgyz republic*':ab,ti OR 'lao pdr*':ab,ti OR lebanon*:ab,ti OR lesotho*:ab,ti OR liberia*:ab,ti OR libya*:ab,ti OR madagascar*:ab,ti OR malawi*:ab,ti OR malaysia*:ab,ti OR maldives*:ab,ti OR mali*:ab,ti OR 'marshall islands*':ab,ti OR mauritania*:ab,ti OR mauritius*:ab,ti OR mexico*:ab,ti OR micronesia*:ab,ti OR moldova*:ab,ti OR mongolia*:ab,ti OR montenegro*:ab,ti OR morocco*:ab,ti OR mozambique*:ab,ti OR myanmar*:ab,ti OR namibia*:ab,ti OR nauru*:ab,ti OR nepal*:ab,ti OR nicaragua*:ab,ti OR niger*:ab,ti OR nigeria*:ab,ti OR 'north macedonia*':ab,ti OR pakistan*:ab,ti OR 'papua new guinea*':ab,ti OR paraguay*:ab,ti OR peru*:ab,ti OR phillipines*:ab,ti OR principe*:ab,ti OR romania*:ab,ti OR rhodesia*:ab,ti OR rwanda*:ab,ti OR samoa*:ab,ti OR 'sao tome*':ab,ti OR senegal*:ab,ti OR 'sierra leone*':ab,ti OR 'solomon islands*':ab,ti OR somalia*:ab,ti OR 'south africa*':ab,ti OR 'southern africa*':ab,ti OR 'south sudan*':ab,ti OR 'sri lanka*':ab,ti OR sudan*:ab,ti OR	

No.	Query	Results
	swaziland*:ab,ti OR syria*:ab,ti OR tajikistan*:ab,ti OR tanzania*:ab,ti OR togo*:ab,ti OR tonga*:ab,ti OR tunisia*:ab,ti OR tunis*:ab,ti OR uganda*:ab,ti OR uzbekistan*:ab,ti OR vanuata*:ab,ti OR venezuela*:ab,ti OR vietnam*:ab,ti OR 'west bank':ab,ti OR gaza*:ab,ti OR 'west africa*':ab,ti OR yemen*:ab,ti OR zaire*:ab,ti OR zambia*:ab,ti OR zimbabwe*:ab,ti) AND [2000-2020]/py	
#2	('communicable disease'/exp/mj OR 'communicable diseases':ab,ti OR 'communicable infection*':ab,ti OR 'communicable chronic disease*':ab,ti OR 'transmittable disease*':ab,ti OR 'transferrable disease*':ab,ti OR 'contaminating disease*':ab,ti OR 'infective disease*':ab,ti OR 'contagious disease*':ab,ti OR 'hiv/aids' OR hiv:ab,ti OR aids:ab,ti OR 'wasting disease*':ab,ti OR 'wasting syndrome':ab,ti OR 'immunodeficiency virus*':ab,ti OR 'virulent disease*':ab,ti OR 'transmissible disease*':ab,ti OR 'transmissible infection*':ab,ti OR 'tuberculosis'/exp/mj OR tuberculos*:ab,ti OR 'tuberculosis infection*':ab,ti OR tb:ab,ti OR t.b.:ab,ti OR consumption*:ab,ti OR 'white plague*':ab,ti OR 'potts disease*':ab,ti OR 'bacilli infection*':ab,ti OR pneumonia*:ab,ti OR phthisis*:ab,ti OR phthysic*:ab,ti OR 'co infection*':ab,ti OR 'mixed infection*':ab,ti OR 'malaria'/exp/mj OR malaria*:ab,ti OR 'plasmodium infection*':ab,ti OR 'ectoparasitic infection*':ab,ti OR ectoparasite*:ab,ti OR 'hepatitis'/exp/mj OR hepatitis:ab,ti OR 'liver disease*':ab,ti OR 'liver infection*':ab,ti OR 'infectious disease*':ab,ti OR 'hepatitis delta*':ab,ti OR 'delta hepatitis*':ab,ti OR 'hepatitis virus*':ab,ti OR 'hepatitis b'/exp/mj OR 'hepatitis b*':ab,ti OR 'infectious hepatitis b*':ab,ti OR 'serum hepatitis*':ab,ti OR 'acute hepatitis b*':ab,ti OR 'acute serum hepatitis*':ab,ti OR 'hepatitis c'/exp/mj OR 'hepatitis c*':ab,ti OR 'peptic ulcer disease'/exp/mj OR 'peptic ulcer disease*':ab,ti OR 'peptic ulcer*':ab,ti OR 'peptic ulceration*':ab,ti OR 'active peptic ulceration*':ab,ti OR 'gastric ulcer*':ab,ti OR gastritis*:ab,ti OR 'gastric infection*':ab,ti OR 'gastric disease*':ab,ti OR 'duodenal ulcer*':ab,ti OR 'duodenal infection*':ab,ti OR 'duodenal disease*':ab,ti OR ulcers*:ab,ti OR 'pyloric infection*':ab,ti OR 'pyloric disease*':ab,ti) AND [2000-2020]/py	**1456312**
#1	('non communicable disease'/exp/mj OR 'non-communicable diseases*':ab,ti OR 'noncommunicable disease*':ab,ti OR 'noninfectious disease*':ab,ti OR 'non infectious disease*':ab,ti OR 'non commmunicable chronic disease*':ab,ti OR 'noncommunicable chronic disease*':ab,ti OR 'cardiovascular disease'/exp/mj OR 'cardiovascular disease*':ab,ti OR 'heart disease*':ab,ti OR 'heart failure*':ab,ti OR 'coronary infarction*':ab,ti OR 'cardiac infarction*':ab,ti OR 'heart infarction*':ab,ti OR 'myocardial infarction*':ab,ti OR 'lung disease, obstructive' OR 'chronic obstructive	**7247401**

No.	Query	Results
	disease*':ab,ti OR asthma*:ab,ti OR emphysema*:ab,ti OR 'chronic obstructive pulmonary disease':ab,ti OR copd*:ab,ti OR 'cystic fibrosis':ab,ti OR bronchitis:ab,ti OR 'neoplasms'/exp/mj OR neoplasm*:ab,ti OR cancer*:ab,ti OR cancers*:ab,ti OR tumor*:ab,ti OR tumour*:ab,ti OR malignancy*:ab,ti OR malignant*:ab,ti OR 'malignant neoplasm*':ab,ti OR 'malignant tumour*':ab,ti OR 'diabetes mellitus'/exp/mj OR diabetes*:ab,ti OR diabetic*:ab,ti OR 'polygenic disease*':ab,ti OR 'polygenic disorder*':ab,ti OR 'mental disorders'/exp/mj OR 'mental disorders*':ab,ti OR 'mental disorder*':ab,ti OR depression*:ab,ti OR 'depressive disorders*':ab,ti OR 'personality disorder*':ab,ti OR 'hypertension'/exp/mj OR hypertension:ab,ti OR 'hypertensive disorder*':ab,ti OR hypertensive*:ab,ti OR 'high blood pressure*':ab,ti) AND [2000-2020]/py	

3. COCHRANE

Search Name: Combined search with publication dates applied

Date Run: 24/02/2020 16:45:17

Comment:

ID Search Hits

#1 (*Noncommunicable Diseases*) OR (Non-communicable disease*):ti,ab OR (Noncommunicable Disease*):ti,ab OR (Noninfectious Disease*):ti,ab OR (Non-infectious Disease*):ti,ab OR (non-communicable chronic disease*):ti,ab OR (noncommunicable-chronic disease*):ti,ab OR (Cardiovascular Diseases*):ti,ab OR (cardiovascular disease*):ti,ab OR (Lung Diseases, Obstructive*):ti,ab OR (Lung Diseases*):ti,ab OR (chronic obstructive*):ti,ab OR (asthma*):ti,ab OR (emphysema):ti,ab OR (COPD*):ti,ab OR (CF*):ti,ab OR (cystic fibrosis*):ti,ab OR (bronchitis*):ti,ab OR (Neoplasms*):ti,ab OR (neoplasm*):ti,ab OR (neoplasms*):ti,ab OR (cancer*):ti,ab OR (cancers*):ti,ab OR (tumor*):ti,ab OR (tumors*):ti,ab OR (tumour*):ti,ab OR (tumours*):ti,ab OR (malignant*):ti,ab OR (malignancy*):ti,ab OR (malignancies*):ti,ab OR (Diabetes Mellitus*):ti,ab OR (diabetes*):ti,ab OR (diabetic*):ti,ab OR (Mental Disorder*):ti,ab OR (Mental disorders*):ti,ab OR (Mental Disorder*):ti,ab OR (Hypertension*):ti,ab OR (Hypertension*):ti,ab
390200

#2 (*Comorbidity*) OR (comorbidity*):ti,ab OR (comorbidit*):ti,ab OR (polymorbidit*):ti,ab OR (*Multiple Chronic*) OR (multiple chronic medical conditions*):ti,ab OR (multiple morbidit*):ti,ab OR (multiple chronic health condition*):ti,ab OR (multimorbidit*):ti,ab OR (multi-morbidit*):ti,ab
32821

#3 (*Communicable disease*) OR (communicable diseases*):ti,ab OR (communicable infection*):ti,ab OR (communicable chronic disease*):ti,ab OR (transmittable disease*):ti,ab OR (transferrable disease*):ti,ab OR (contaminating disease*):ti,ab OR (infective disease*):ti,ab OR (contagious disease*):ti,ab OR (hivaids*):ti,ab OR (hiv*):ti,ab OR (aids*):ti,ab OR (wasting disease*):ti,ab OR (wasting syndrome*):ti,ab OR (immunodeficiency virus*):ti,ab OR (virulent disease*):ti,ab OR (transmissible disease*):ti,ab OR (transmissible infection*):ti,ab OR (Tuberculosis*):ti,ab OR (tuberculos*):ti,ab OR (tuberculosis infection*):ti,ab OR (tb*):ti,ab OR (t.b.*):ti,ab OR (consumption*):ti,ab OR (white plague*):ti,ab OR (potts disease*):ti,ab OR (bacilli infection*):ti,ab OR (pneumonia*):ti,ab OR (phthisis*):ti,ab OR (phthysic*):ti,ab OR (co-infection*):ti,ab OR (mixed infection*):ti,ab OR (Malaria*):ti,ab OR (malaria*):ti,ab OR (plasmodium infection*):ti,ab OR (ectoparasitic infection*):ti,ab OR (ectoparasite*):ti,ab OR (hepatitis*):ti,ab OR (liver disease*):ti,ab OR (liver infection*):ti,ab OR (infectious disease*):ti,ab OR (hepatitis delta*):ti,ab OR (delta hepatitis*):ti,ab OR (hepatitis virus*):ti,ab OR (hepatitis b*):ti,ab OR (hepatitis b*):ti,ab OR (infectious hepatitis b*):ti,ab OR (serum hepatitis*):ti,ab OR (acute hepatitis b*):ti,ab OR (acute serum hepatitis*):ti,ab OR (hepatitis c*):ti,ab OR (hepatitis c*):ti,ab OR (peptic ulcer disease*) OR (peptic ulcer disease*):ti,ab OR (peptic ulcer*):ti,ab OR (peptic ulceration*):ti,ab OR (active peptic ulceration*):ti,ab OR (gastric ulcer *):ti,ab OR (gastritis*):ti,ab OR (gastric infection*):ti,ab OR (gastric disease*):ti,ab OR (duodenal ulcer*):ti,ab OR (duodenal infection*):ti,ab OR (duodenal disease*):ti,ab OR (ulcers*):ti,ab OR (pyloric infection*):ti,ab OR (pyloric disease*):ti,ab 155376

#4 (*Developing Countries OR (developing countr*):ti,ab OR (developing nation*):ti,ab OR (developing population*):ti,ab OR (developing econom*):ti,ab OR (undeveloped countr*):ti,ab OR (undeveloped nation*):ti,ab OR (undeveloped econom*):ti,ab OR (least developed countr*):ti,ab OR (least developed nation*):ti,ab OR (least developed econom*):ti,ab OR (least developed econom*):ti,ab OR (less developed countr*):ti,ab OR (less developed nation*):ti,ab OR (less developed population*):ti,ab OR (less developed econom*):ti,ab OR (lesser developed countr*):ti,ab OR (lesser developed nation*):ti,ab OR (lesser developed population*):ti,ab OR (lesser developed econom*):ti,ab OR (under developed countr*):ti,ab OR (under developed nation*):ti,ab OR (under developed countr*):ti,ab OR (under developed nation*):ti,ab OR (underdeveloped population*):ti,ab OR (underdeveloped econom*):ti,ab OR (low income countr*):ti,ab OR (middle income countr*):ti,ab OR (low income nation*):ti,ab OR (middle income nation*):ti,ab OR (low income population*):ti,ab OR (middle income population*):ti,ab OR (low income econom*):ti,ab OR (middle income econom*):ti,ab OR (lower income countr*):ti,ab OR (lower income nation*):ti,ab OR (lower income population*):ti,ab OR (lower income econom*):ti,ab OR (lower income econom*):ti,ab OR (resource limited*):ti,ab OR low resource countr*):ti,ab OR (lower resource countr*):ti,ab OR (low resource nation*):ti,ab OR (low resource population*):ti,ab OR (low resource econom*):ti,ab OR (low resource econom*):ti,ab OR (underserved countr*):ti,ab OR (underserved nation*):ti,ab OR (underserved population*):ti,ab OR (underserved econom*):ti,ab OR (under served countr*):ti,ab OR (under served population*):ti,ab OR (under served nation*):ti,ab OR (derived countr*):ti,ab OR (deprived nation*):ti,ab OR (deprived nation*):ti,ab OR (derived population*):ti,ab OR (deprived econom*):ti,ab OR (poor countr*):ti,ab OR (poor nation*):ti,ab OR (poor population*):ti,ab OR (poor econom*):ti,ab OR (poorer countr*):ti,ab OR (poorer nation*):ti,ab OR (poorer population*):ti,ab OR (poorer econom*):ti,ab OR (lmic*):ti,ab OR (lmics*):ti,ab OR (transitional countr*):ti,ab OR (transitional nation*):ti,ab OR (transitional econom*):ti,ab OR (transition countr*):ti,ab OR (transition nation*):ti,ab OR (transition econom*):ti,ab OR (low resource setting*):ti,ab OR (lower

resource setting*):ti,ab OR (middle resource setting*):ti,ab OR (Third world*):ti,ab OR (South East Asia*):ti,ab OR (Middle east*):ti,ab OR (Afghan*):ti,ab OR (Angola*):ti,ab OR (Angolese*):ti,ab OR (Angolian*):ti,ab OR (Armenia*):ti,ab OR (Albania*):ti,ab OR (Algeria*):ti,ab OR (Bangladesh*):ti,ab OR (Benin*):ti,ab OR (Bhutan*):ti,ab OR (Birma*):ti,ab OR (Burma*):ti,ab OR (Birmese*):ti,ab OR (Burmese*):ti,ab OR (Bolivia*):ti,ab OR (Botswana*):ti,ab OR (Burkina Faso*):ti,ab OR (Burundi*):ti,ab OR (Cabo Verde*):ti,ab OR (Cambodia*):ti,ab OR (Cameroon*):ti,ab OR (Central Africa*):ti,ab OR (Chad*):ti,ab OR (Comoros*):ti,ab OR (Congo*):ti,ab OR (Cote dIvoire*):ti,ab OR (Djibouti*):ti,ab OR (East Africa*):ti,ab OR (Eastern Africa*):ti,ab OR (Egypt*):ti,ab OR (El Salvador*):ti,ab OR (Equatorial Guinea*):ti,ab OR (Eritrea*):ti,ab OR (Ethiopia*):ti,ab OR (Gabon*):ti,ab OR (Gambia*):ti,ab OR (Gaza*):ti,ab OR (Georgia Republic*):ti,ab OR (Grenada*):ti,ab OR (Ghana*):ti,ab OR (Guatemala*):ti,ab OR (Guinea*):ti,ab OR (Haiti*):ti,ab OR (India*):ti,ab OR (Indonesia*):ti,ab OR (Ivory Coast*):ti,ab OR (Kenya*):ti,ab OR (Kiribati*):ti,ab OR (Kosovo*):ti,ab OR (Kyrgyz republic*):ti,ab OR (Lao PDR*):ti,ab OR (Laos*):ti,ab OR (Lesotho*):ti,ab OR (Liberia*):ti,ab OR (Madagascar*):ti,ab OR (Malawi*):ti,ab OR (Mali*):ti,ab OR (Mauritania*):ti,ab OR (Mauritia*):ti,ab OR (Micronesia*):ti,ab OR (Mozambique*):ti,ab OR (Moldovo*):ti,ab OR (Mongolia*):ti,ab OR (Morocco*):ti,ab OR (Mozambique*):ti,ab OR (Myanmar*):ti,ab OR (Namibia*):ti,ab OR (Nepal*):ti,ab OR (Nicaragua*):ti,ab OR (Niger*):ti,ab OR (North Korea*):ti,ab OR (Northern Korea*):ti,ab OR (Democratic Peoples Republic of Korea*):ti,ab OR (Pakistan*):ti,ab OR (Papua New Guinea*):ti,ab OR (Philippine*):ti,ab OR (Principe*):ti,ab OR (Rhodesia*):ti,ab OR (Rwanda*):ti,ab OR (Samoa*):ti,ab OR (Sao Tome*):ti,ab OR (Senegal*):ti,ab OR (Sierra Leone*):ti,ab OR (Solomon Islands*):ti,ab OR (Somalia*):ti,ab OR (South Africa*):ti,ab OR (South Sudan*):ti,ab OR (Southern Africa*):ti,ab OR (Sri Lanka*):ti,ab OR (Sub Saharan Africa*):ti,ab OR (Sudan*):ti,ab OR (Swaziland*):ti,ab OR (Syria*):ti,ab OR (Tajikistan*):ti,ab OR (Tanzania*):ti,ab OR (Timor*):ti,ab OR (Togo*):ti,ab OR (Tonga*):ti,ab OR (Tunis*):ti,ab OR (Uganda*):ti,ab OR (Ukraine*):ti,ab OR (Uzbekistan*):ti,ab OR (Vanuatu*):ti,ab OR (Venezuela*):ti,ab OR (Vietnam*):ti,ab OR (West Africa*):ti,ab OR (West Bank*):ti,ab OR (Western Africa*):ti,ab OR (Yemen*):ti,ab OR (Zaire*):ti,ab OR (Zambia*):ti,ab OR (Zimbabwe*):ti,ab 90632#5 #1 AND #2 AND #3 AND #4 with Cochrane Library publication date Between Jan 2000 and Jan 2020 611

Section and Topic	Item #	Checklist item
**TITLE**		
Title	1	Identify the report as a systematic review.
**ABSTRACT**		
Abstract	2	See the PRISMA 2020 for Abstracts checklist.
**INTRODUCTION**		
Rationale	3	Describe the rationale for the review in the context of existing knowledge.
Objectives	4	Provide an explicit statement of the objective(s) or question(s) the review addresses.
**METHODS**		
Eligibility criteria	5	Specify the inclusion and exclusion criteria for the review and how studies were grouped for the synthes

Section and Topic	Item #	Checklist item
Information sources	6	Specify all databases, registers, websites, organisations, reference lists and other sources searched or the date when each source was last searched or consulted.
Search strategy	7	Present the full search strategies for all databases, registers and websites, including any filters and limit
Selection process	8	Specify the methods used to decide whether a study met the inclusion criteria of the review, including h record and each report retrieved, whether they worked independently, and if applicable, details of auton
Data collection process	9	Specify the methods used to collect data from reports, including how many reviewers collected data fro independently, any processes for obtaining or confirming data from study investigators, and if applicabl the process.
Data items	10a	List and define all outcomes for which data were sought. Specify whether all results that were compatib study were sought (e.g. for all measures, time points, analyses), and if not, the methods used to decide
	10b	List and define all other variables for which data were sought (e.g. participant and intervention characte assumptions made about any missing or unclear information.
Study risk of bias assessment	11	Specify the methods used to assess risk of bias in the included studies, including details of the tool(s) u each study and whether they worked independently, and if applicable, details of automation tools used i
Effect measures	12	Specify for each outcome the effect measure(s) (e.g. risk ratio, mean difference) used in the synthesis c
Synthesis methods	13a	Describe the processes used to decide which studies were eligible for each synthesis (e.g. tabulating th and comparing against the planned groups for each synthesis (item #5)).
	13b	Describe any methods required to prepare the data for presentation or synthesis, such as handling of m conversions.
	13c	Describe any methods used to tabulate or visually display results of individual studies and syntheses.
	13d	Describe any methods used to synthesize results and provide a rationale for the choice(s). If meta-analy model(s), method(s) to identify the presence and extent of statistical heterogeneity, and software packa
	13e	Describe any methods used to explore possible causes of heterogeneity among study results (e.g. subg
	13f	Describe any sensitivity analyses conducted to assess robustness of the synthesized results.
Reporting bias assessment	14	Describe any methods used to assess risk of bias due to missing results in a synthesis (arising from rep
Certainty assessment	15	Describe any methods used to assess certainty (or confidence) in the body of evidence for an outcome.
**RESULTS**		
Study selection	16a	Describe the results of the search and selection process, from the number of records identified in the se in the review, ideally using a flow diagram.
	16b	Cite studies that might appear to meet the inclusion criteria, but which were excluded, and explain why t
Study characteristics	17	Cite each included study and present its characteristics.
Risk of bias in studies	18	Present assessments of risk of bias for each included study.
Results of individual studies	19	For all outcomes, present, for each study: (a) summary statistics for each group (where appropriate) an precision (e.g. confidence/credible interval), ideally using structured tables or plots.
Results of syntheses	20a	For each synthesis, briefly summarise the characteristics and risk of bias among contributing studies.
	20b	Present results of all statistical syntheses conducted. If meta-analysis was done, present for each the s (e.g. confidence/credible interval) and measures of statistical heterogeneity. If comparing groups, descri
	20c	Present results of all investigations of possible causes of heterogeneity among study results.
	20d	Present results of all sensitivity analyses conducted to assess the robustness of the synthesized results
Reporting biases	21	Present assessments of risk of bias due to missing results (arising from reporting biases) for each synth
Certainty of evidence	22	Present assessments of certainty (or confidence) in the body of evidence for each outcome assessed.
**DISCUSSION**		
Discussion	23a	Provide a general interpretation of the results in the context of other evidence.
	23b	Discuss any limitations of the evidence included in the review.

Section and Topic	Item #	Checklist item
	23c	Discuss any limitations of the review processes used.
	23d	Discuss implications of the results for practice, policy, and future research.
**OTHER INFORMATION**		
Registration and protocol	24a	Provide registration information for the review, including register name and registration number, or state
	24b	Indicate where the review protocol can be accessed, or state that a protocol was not prepared.
	24c	Describe and explain any amendments to information provided at registration or in the protocol.
Support	25	Describe sources of financial or non-financial support for the review, and the role of the funders or spon
Competing interests	26	Declare any competing interests of review authors.
Availability of data, code and other materials	27	Report which of the following are publicly available and where they can be found: template data collectio studies; data used for all analyses; analytic code; any other materials used in the review.

From: Page MJ, McKenzie JE, Bossuyt PM, Boutron I, Hoffmann TC, Mulrow CD, et al. The PRISMA 2020 statement: an updated guideline for reporting systematic reviews. BMJ 2021;372:n71. doi: 10.1136/bmj.n71

For more information, visit: http://www.prisma-statement.org/
